# A phase II, multicentre, UK study of vinorelbine in advanced breast cancer.

**DOI:** 10.1038/bjc.1994.435

**Published:** 1994-11

**Authors:** C. J. Twelves, N. A. Dobbs, A. Curnow, R. E. Coleman, A. L. Stewart, C. J. Tyrrell, P. Canney, R. D. Rubens

**Affiliations:** Imperial Cancer Research Fund Clinical Oncology Unit, UMDS, Guy's Hospital, London, UK.

## Abstract

Thirty-four evaluable patients were treated with vinorelbine, a novel, semisynthetic vinca alkaloid, as first-line chemotherapy for advanced breast cancer. They received vinorelbine 25 mg m-2 i.v. given weekly for a maximum of 16 cycles. Two patients achieved a complete remission and 15 a partial remission, giving a response rate of 17/34 (50%; 95% CI of 34-66%); median response duration was 5.8 months. The median progression-free interval was 4.4 months and median survival 9.9 months. Treatment was generally well tolerated. Fatigue was the most common side-effect. The main reason for dose adjustments was myelosuppression; 68% of patients had WHO grade 3 or 4 neutropenia and there was one death attributed to neutropenic sepsis. Nausea/vomiting and neuropathy were mild and alopecia was uncommon. This study confirms vinorelbine as a highly active, well-tolerated agent in advanced breast cancer worthy of evaluation in combination chemotherapy regimens.


 © Macmillan Press Ltd., 1994

### A phase II, multicentre, UK study of vinorelbine in advanced breast cancer

C.J. Twelves[1], N.A. Dobbs[1], A. Curnow[1], R.E. Coleman[2], A.L. Stewart[3], C.J. Tyrrell[4], P. Canney[5] & R.D. Rubens[1]

[1]*Imperial Cancer Research Fund Clinical Oncology Unit, UMDS, Guy's Hospital, St Thomas Street, London SE1 9RT, UK;* [2]*Yorkshire Cancer Research Campaign Department of Clinical Oncology, Weston Park Hospital, Sheffield, UK;* [3]*The Christie Hospital and Holt Radium Institute, Manchester, UK;* [4]*Department of Radiotherapy and Oncology, Plymouth General Hospital, Freedom Fields, Plymouth, UK;* [5]*Belvidere Hospital, Glasgow, UK.*

**Summary** Thirty-four evaluable patients were treated with vinorelbine, a novel, semisynthetic vinca alkaloid, as first-line chemotherapy for advanced breast cancer. They received vinorelbine 25 mg $m^{-2}$ i.v. given weekly for a maximum of 16 cycles. Two patients achieved a complete remission and 15 a partial remission, giving a response rate of 17/34 (50%; 95% CI of 34–66%); median response duration was 5.8 months. The median progression-free interval was 4.4 months and median survival 9.9 months. Treatment was generally well tolerated. Fatigue was the most common side-effect. The main reason for dose adjustments was myelosuppression; 68% of patients had WHO grade 3 or 4 neutropenia and there was one death attributed to neutropenic sepsis. Nausea/vomiting and neuropathy were mild and alopecia was uncommon. This study confirms vinorelbine as a highly active, well-tolerated agent in advanced breast cancer worthy of evaluation in combination chemotherapy regimens.

Although a wide range of agents are active against breast cancer, the treatment of advanced disease is palliative not curative. There remains a need for new agents with either greater activity or reduced toxicity compared with those now in use. The vinca alkaloids were first evaluated in breast cancer during the 1960s. Response rates for vincristine and vinblastine, and later the semisynthetic compound vindesine, were modest and neuropathy significant (Henderson, 1991). As a result, these agents have had only a limited role in the treatment of advanced breast cancer. More recently a new group of compounds, differing in both the vindoline and catharanthine moeities, was synthesised, of which vinorelbine (nor-5′-anhydrovinblastine, Navelbine) has entered clinical trials (Figure 1).

Preclinical studies suggested that vinorelbine may have an improved therapeutic index relative to other vinca alkaloids. In common with other vinca alkaloids vinorelbine acts on the mitotic spindle. However, the effect of vinorelbine on tubulin differs qualitatively and quantitatively from that of vincristine or vinblastine (Fellous *et al.*, 1989). Inhibition of tubulin polmerisation is greater, but induced spiralisation less, with vinorelbine than with vinblastine. Vinorelbine has activity against a wide range of murine tumours, including L1210 leukaemia, P388 leukaemia and B16 melanoma. It is also effective against human lung and stomach xenografts (Cros *et al.*, 1989). In the phase I clinical study by Mathe and Reizenstein (1985) neutropenia was the dose-limiting toxicity in heavily pretreated patients. Neurotoxicity was mild, although many patients had previously received other vinca alkaloids. When the current study opened in 1992, preliminary reports of phase II studies suggested that vinorelbine 30 mg $m^{-2}$ given weekly had substantial activity in heavily pretreated patients (Marty *et al.*, 1989) and as first-line chemotherapy for advanced breast cancer (Canobbio *et al.*, 1989; Fumoleau *et al.*, 1990; Mickiewicz *et al.*, 1991).

Prior to the current study vinorelbine had not been evaluated in the UK. The aim of this phase II study was to determine the efficacy and toxicity of vinorelbine 25 mg $m^{-2}$ given weekly to patients with advanced breast cancer at centres within this country. Previous studies evaluating a dose of 30 mg $m^{-2}$ had required frequent dose modifications (Canobbio *et al.*, 1989; Fumoleau *et al.*, 1990; Mickiewicz *et al.*, 1991). A slightly lower dose was selected for the current study in an attempt to maintain the planned dose intensity without sacrificing efficacy.

Correspondence: C.J. Twelves.
Received 5 May 1994; and in revised form 5 July 1994.

### Patients and methods

### *Patients*

Eligible patients had histologically confirmed advanced breast cancer with at least one bidimensionally measurable lesion. They had not received prior chemotherapy for advanced disease but previous adjuvant chemotherapy was permitted provided there had been a 6 month disease-free period after finishing such treatment. Prior endocrine treatment and radiotherapy were allowed. Other eligibility criteria included:

| *Compound* | *R₁* | *R₂* |
|---|---|---|
| Vinorelbine | – | $CH_3$ |
| Vinblastine | OH* | $CH_3$ |
| Vincristine | OH* | CHO |

*Vinblastine and vincristine have no double bond at $R_1$.

**Figure 1** Structure of the vinca alkaloids.

WHO performance status 0–2; age 18–75 years; white blood cells > $3.0 \times 10^9 l^{-1}$, neutrophils > $2.0 \times 10^9 l^{-1}$ and platelets > $100 \times 10^9 l^{-1}$; serum bilirubin, serum transaminases and creatinine < 1.25 times the upper limit of the reference range (unless the abnormalities were directly attributable to breast cancer). Patients with clinical signs of peripheral neuropathy (unless directly attributable to malignancy), brain metastases or a previous history of other malignancy were excluded.

Treatment toxicity was assessed according to WHO (1979) criteria and response by UICC guidelines (Hayward *et al.*, 1981). Bone metastases were not accepted as the sole site of evaluable disease. They were assessed only if lytic and were then considered evaluable but not measurable. The protocol specified a response rate of 20% as the lowest that may indicate significant treatment efficacy. Response duration, progression-free interval and survival were measured from the date of the first cycle of vinorelbine (Kaplan & Meier, 1958).

All patients gave informed, written consent and the study was approved by the ethics committee of each of the participating hospitals.

### *Treatment plan*

Patients received vinorelbine as an intravenous bolus injection over 3–5 min followed by flushing with saline. Treatment was given weekly, initially at a dose of $25 \text{ mg m}^{-2}$. This was reduced to $20 \text{ mg m}^{-2}$ if subsequently the neutrophils were $1.0–1.5 \times 10^9 l^{-1}$ or platelets $75–100 \times 10^9 l^{-1}$. Treatment was delayed if the blood count was lower than these values. Patients who developed neurotoxicity of WHO grade 2 or worse had treatment delayed until this recovered. If neurotoxicity persisted for more than 4 weeks treatment was to be discontinued. Patients who responded could receive a maximum of 16 weekly cycles of vinorelbine; those with stable disease received a maximum of eight treatments. Most patients routinely received metoclopramide 10 mg i.v. alone as antiemetic cover. Treatment at relapse was at the discretion of the clinician.

**Table I** Characteristics of evaluable patients ($n$ = 34)

| | |
|---|---|
| Median age (years) | 59 (range 34–75) |
| Sex | |
| Female | 33 (6 pre- and 27 post-menopausal) |
| Male | 1 |
| Median time from diagnosis to vinorelbine (months) | 24 (range 0–187) |
| ECOG performance status: | |
| 0 | 13 |
| 1 | 16 |
| 2 | 5 |
| Histology | |
| Infiltrating ductal | 21 |
| Infiltrating lobular | 5 |
| Other/unknown | 8 |
| Receptor status: | |
| ER positive | 9 |
| ER negative | 0 |
| Unknown | 25 |
| PR positive | 7 |
| PR negative | 2 |
| Unknown | 25 |
| Prior systemic treatment: | |
| Adjuvant endocrine | 18 |
| Adjuvant chemotherapy | 3 |
| Advanced endocrine | 19 |
| Measurable/evaluable disease sites:[a] | |
| Cutaneous | 11 |
| Lymphatic | 22 |
| Breast/local recurrence | 9 |
| Soft tissue | 4 |
| Bone | 7 |
| Visceral | 18 (12 lung, six liver) |
| Other | 5 (three ascites, one pleural, one abdominal) |

[a]Eight patients had one, 13 patients had two and 13 patients had three or more sites of measurable/evaluable disease

**Table II** Response to vinorelbine by disease site

| | Responses (%) | | |
|---|---|---|---|
| *Disease site (n)* | *CR* | *PR* | *CR + PR* |
| Skin (11) | 4 | 3 | 7 (64) |
| Lymph nodes (22) | 6 | 5 | 11 (50) |
| Breast local recurrence (9) | 1 | 2 | 3 (33) |
| Soft tissue (4) | 1 | 2 | 3 (75) |
| Bone (7) | 1 | 1 | 2 (29) |
| Visceral (18) | 2 | 6 | 8 (44) |
| Other (5) | 1 | 0 | 1 (20) |
| Total (76) | 16 | 19 | 35 (46) |

### Results

A total of 35 patients entered the study between April and December 1992. One patient, who was ineligible since she remained on tamoxifen, was excluded from the analyses. The pretreatment characteristics of the remaining 34 eligible patients, one of whom was male, are shown in Table I.

These patients received a total of 354 cycles of vinorelbine; the median number of cycles administered was 10 (range 3–16). Nine patients completed the full course of 16 cycles of vinorelbine and 13 stopped because of progressive disease. Of the remaining patients, five declined further treatment and six stopped at their physician's discretion (two with a partial remission and four with stable disease); there was one toxic death. The median dose–time treatment intensity was 79% of that planned (range 55–99%). Dose adjustments were made for 129 (36%) cycles of vinorelbine; 53 (15%) cycles were given at a reduced dose, 61 (17%) were delayed and 15 (4%) were both delayed and given at reduced dose. Neutropenia accounted for most dose adjustments 98/129 (76%).

All 34 patients were evaluable for treatment efficacy and toxicity with a median follow-up of 18.2 months. Two patients achieved a complete response (CR), 15 a partial response (PR), 12 stable disease and five had progressive disease. The response rate was, therefore, 50% with 95% confidence intervals (CI) of 34–66%; median response duration was 5.8 months (range 2.3–9.8 months). The male patient did not respond to vinorelbine. As Table II shows, responses were seen at each of the sites of disease. In all, 35 responses were seen from a total of 76 measurable or evaluable disease sites (46%). The median time to progression for all 34 patients was 4.4 months (range 0.9 to > 14.4 months); median survival was 9.9 months (range 1.8 to > 21.1 months).

Haematological toxicity is shown in Table III and other WHO toxicities in Table IV. Neutropenia was the main toxicity. WHO grade 3 or 4 neutropenia was experienced by 12 patients and 11 patients respectively; this occurred in 16% of treatment cycles. There was one toxic death, attributed to neutropenic sepsis, in a woman who died 5 days after her 12th cycle of vinorelbine. Contrary to the protocol this had been given at full dose, rather than reduced to $20 \text{ mg m}^{-2}$, despite a neutrophil count of $1.3 \times 10^9 l^{-1}$. This patient received two previous cycles of treatment without the dose modifications recommended in the protocol. Significant anaemia was very uncommon, with only one patient developing WHO grade 3 toxicity for a single cycle of vinorelbine. Thrombocytopenia was not observed.

Non-haematological toxicity was generally mild. Asthenia (tiredness), which is not graded by WHO criteria, was the most common symptom during treatment. Mild, moderate

**Table III** Maximum haematological toxicity

| | *Number of patients WHO grade* | | | | | *Number of cycles*[a] *WHO grade* | | | | |
|---|---|---|---|---|---|---|---|---|---|---|
| *Toxicity* | *0* | *1* | *2* | *3* | *4* | *0* | *1* | *2* | *3* | *4* |
| Neutropenia | 2 | 2 | 7 | 12 | 11 | 172 | 50 | 62 | 37 | 18 |
| | | | | 68% | | | | | 16% | |
| Leucopenia | 0 | 5 | 11 | 16 | 2 | 130 | 85 | 84 | 41 | 3 |
| | | | | 53% | | | | | 13% | |
| Thrombocytopenia | 34 | 0 | 0 | 0 | 0 | 343 | 0 | 0 | 0 | 0 |
| | | | | 0% | | | | | 0% | |
| Anaemia | 9 | 18 | 6 | 1 | 0 | 190 | 129 | 23 | 1 | 0 |
| | | | | 3% | | | | | <1% | |

[a]$n$ = 343 evaluable cycles, except for neutrophils, where $n$ = 339 cycles.

**Table IV** Maximum non-haematological toxicity

| | *Number of patients WHO grade* | | | | | *Number of cycles*[a] *WHO grade* | | | | |
|---|---|---|---|---|---|---|---|---|---|---|
| *Toxicity* | *0* | *1* | *2* | *3* | *4* | *0* | *1* | *2* | *3* | *4* |
| Nausea/vomiting | 10 | 12 | 8 | 4 | 0 | 240 | 79 | 22 | 4 | 0 |
| | | | | 11% | | | | | 1% | |
| Neuropathy | 24 | 7 | 3 | 0 | 0 | 284 | 54 | 7 | 0 | 0 |
| | | | | 0% | | | | | 0% | |
| Alopecia | 10 | 13 | 7 | 4 | 0 | 213 | 78 | 47 | 7 | 0 |
| | | | | 12% | | | | | 2% | |
| Constipation | 11 | 10 | 11 | 2 | 0 | 269 | 47 | 26 | 3 | 0 |
| | | | | 6% | | | | | <1% | |
| Diarrhoea | 24 | 4 | 5 | 1 | 0 | 322 | 15 | 7 | 1 | 0 |
| | | | | 3% | | | | | <1% | |
| Infection | 17 | 13 | 2 | 1 | 1 | 320 | 22 | 2 | 1 | 1 |
| | | | | 6% | | | | | <1% | |
| Phlebitis | 19 | 8 | 6 | 1 | 0 | 296 | 34 | 13 | 2 | 0 |
| | | | | 3% | | | | | <1% | |
| Stomatitis | 20 | 10 | 4 | 0 | 0 | 311 | 28 | 6 | 0 | 0 |
| | | | | 0% | | | | | <0% | |

[a]$n$ = evaluable 345 cycles, except for infection, where $n$ = 346 cycles.

and severe asthenia were reported with 26%, 24% and 3.5% respectively of cycles of vinorelbine; these degress of asthenia were experienced by 29%, 26% and 6% patients respectively. Table IV shows the other non-haematological WHO toxicities. Infection was uncommon and WHO grade 3 and 4 infection associated with only two cycles of treatment in two patients, one of whom died. Peripheral neuropathy was uncommon and mild; the worst neuropathy experienced was grade II in three patients. Although the majority of patients received metoclopramide as the only antiemetic, most cycles of chemotherapy were associated with no clinically significant nausea and vomiting. Severe (WHO grade 3) vomiting was recorded in 4% of patients, although this represented only 1% of treatment cycles. Alopecia was mild and stomatitis was uncommon.

### Discussion

The preliminary results of several studies evaluating vinorelbine given as a single agent in patients with advanced breast cancer have been reported (Marty *et al.*, 1989; Canobbio *et al.*, 1989; Fumoleau *et al.*, 1990; Mickiewicz *et al.*, 1991; Lluch *et al.*, 1992). However, so far the final results of only two studies have been published (Fumoleau *et al.*, 1993; Romero *et al.*, 1994). It is valuable to evaluate a new agent in different populations since treatment principles may differ between countries. The most important finding of this phase II study conducted at several centres in the UK is to confirm that vinorelbine has considerable activity, and is well tolerated, in patients with advanced breast cancer.

There are differences between the current study and the previous published phase II trials in patients with advanced breast cancer (Fumoleau *et al.*, 1993; Romero *et al.*, 1994). Firstly, this is the first study to have evaluated the dose of 25 mg $m^{-2}$ rather than 30 mg $m^{-2}$. Secondly, patients who responded received a maximum of 16 cycles rather than continuing to disease progression or dose-limiting toxicity as was the case for both the other trials. The response rate of 50% in the current study was very similar to that of 41% reported previously (Fumoleau *et al.*, 1993; Romero *et al.*, 1994). Median time to progression in all patients was 4.4

months in the current study, only slightly less than the 6.0 months reported by Fumoleau *et al.* (1993) and Romero *et al.* (1994). The median response duration in our study was 5.8 months, substantially shorter than that of 7.8 months and 9 months reported by both the earlier studies (Fumoleau *et al.*, 1993; Romero *et al.*, 1994). Similarly, the median survival of 9.7 months in the current study was substantially less than that of 18 months reported previously (Fumoleau *et al.*, 1993). These discrepancies may be the result of differences in the patient populations. However, the pretreatment characteristics of the patients were similar and the median number of treatment cycles given and delivered dose-time intensity were also similar. The longer response duration reported by Fumoleau *et al.* (1993) and Romero *et al.* (1994) may reflect the differing treatment policies whereby patients who responded continued treatment to progression or unacceptable toxicity. A further difference may be better survival after relapse following vinorelbine in the earlier trials (Fumoleau *et al.*, 1993; Romero *et al.*, 1994). This might be explained by differences in the treatment regimens, and their efficacy, between the various study populations.

Vinorelbine was generally well tolerated in the current study. The most consistent toxicity was asthenia or tiredness, which may be drug related or a result of the underlying cancer. Tiredness is not included in WHO toxicity scales and was not assessed by Fumoleau *et al.* (1993) or Romero *et al.* (1994). This emphasises the advantages of using the NCI common toxicity criteria, which do evaluate tiredness, and of including quality of life measures in phase III studies of palliative chemotherapy. Myelosuppression, specifically neutropenia, was the main WHO toxicity. This was responsible for the overwhelming majority of dose adjustments. The single toxic death may have resulted from a clinical decision not to lower the dose to 20 mg $m^{-2}$ in a patient with a neutrophil count of $1.3 \times 10^9 l^{-1}$ as this was only marginally below the dose reduction level. Gastrointestinal toxicity and peripheral neuropathy were mild, and alopecia was uncommon. This pattern of toxicity in the current study was very similar to that described previously (Fumoleau *et al.*, 1993; Romero *et al.*, 1994).

Comparisons between the response rates with vinorelbine and those of other vinca alkaloids are difficult since in the early studies the patient groups were less well defined and standard assessment criteria were often not available. Nevertheless, in a review by Henderson (1991) the response rates quoted for vincristine 19% (CI 15-24%), vinblastine 21% (CI 15-29%) and vindesine 24% (CI 18-30%) appear lower than that for vinorelbine of 43% (95% CI 36-50%) in the three phase II studies now published. The response rates to vinorelbine are similar to those of the most effective single agents in advanced breast cancer (Henderson, 1991). This encouraging activity and low toxicity of vinorelbine given as a single agent have led to studies investigating its activity in combination with other agents as first-line treatment in patients with advanced breast cancer. Preliminary results have been encouraging. Dieras *et al.* (1990) reported a response rate of 63% in patients receiving vinorelbine 30 mg $m^{-2}$ with 5-fluorouracil 750 mg $m^{-2}$ by continuous infusion, each given on days 1 and 5 every 3 weeks. The combination of vinorelbine 25 mg $m^{-2}$ on days 1 and 8 with doxorubicin 50 mg $m^{-2}$ on day 1 achieved a response rate of 78% (Dorval *et al.*, 1991).

This study has confirmed that vinorelbine 25 mg $m^{-2}$ given weekly as a single agent is highly active and well tolerated as first-line treatment in patients with advanced breast cancer. These encouraging phase II data need to be confirmed in phase III studies supported by quality of life data. Vinorelbine may then have an important place in the treatment of patients with advanced breast cancer, as adjuvant treatment in women with early disease or primary chemotherapy for locally advanced disease.

We thank Ms K. King, Ms P. Perry (Farmitalia Carlo Erba), Dr A. Timothy (St Thomas' Hospital) and Dr M.A. Richards (Guy's Hospital) for their help. This study was conducted in collaboration with Farmitalia Carlo Erba and Pierre Fabre Medicament.

### References

CANOBBIO, L., BOCCARDO, F., PASTORINO, G., BREMA, F., MARTINI, C., RENASCO, M. & SANTI, L. (1989). Phase-II study of navelbine in advanced breast cancer. *Semin. Oncol.*, **16** (2) (Suppl. 4), 33-36.

CROS, S., WRIGHT, M., MORIMOTO, M., LATASTE, H., COUZINIER, J.P. & KRIKORIAN, A. (1989). Experimental antitumour activity of navelbine. *Semin. Oncol.*, **16** (2) (Suppl. 4), 15-20.

DIERAS, V., EXTRA, J.M., MORVAN, F., BELLISSANT, E., FANDI, A., ESPIE, M., BADRI, N. & MARTY, M. (1990). Phase II study of navelbine (NVB) and fluorouracil in metastatic breast cancer (MBC) patients using a group sequential design. *Breast Cancer Res. Treat.*, **16**, 161.

DORVAL, T., SPIELMAN, M., SAHRI, C., JOUVE, M., TURPIN, F., ROUESSE, J., POUILLART, T., TURSZ, T. & MERLE, S. (1991). Phase II trial adriamycin and vinorelbine in metastatic breast cancer. *Eur. J. Cancer*, **27** (Suppl. 2), S55 (abstract 304).

FELLOUS, S., OHAYON, T., VACASSIN, T., BINET, S., LATASTE, H., COUZINIER, J.P. & MEININGER, V. (1989). Biochemical effects of navelbine on tubulin and associated proteins. *Semin. Oncol.*, **16** (Suppl. 4), 15-20.

FUMOLEAU, P., DELGADO, F.M., DELOZIER, T., GIL, M.A., BRUNE, C., DANET, S., KERBRAT, P., CHOLLET, P., MONNIER, A., MONNIER, A. & NAMER, M. (1990). Phase II trial with navelbine (NVB) in advanced breast cancer (ABC). *Proc. Am. Soc. Clin. Oncol.*, **9**, 21 (abstract 76).

FUMOLEAU, P., DELGADO, F.M., DELOZIER, T., MONNIER, A., GIL DELGADO, M.A., KERBRAT, P., GARCIA-GIRALT, E., KEILING, R., NAMER, M., CLOSON, M.T., GOUDIER, M.J., CHOLLET, P., LECOURT, L. & MONTCUQUET, ? (1993). Phase II trial of weekly intravenous vinorelbine in first-line advanced breast cancer chemotherapy. *J. Clin. Oncol.*, **11**, 1245-1252.

HAYWARD, J.L., CARBONE, P., HEUSON, J.C., KUMAOKA, S., SEGALOFF, A. & RUBENS, R.D. (1981). Assessment of response in advanced breast cancer. *Eur. J. Cancer*, **13**, 89-94.

HENDERSON, I.C. (1991). Chemotherapy for metastatic disease. In *Breast Diseases*, Harris, J.R., Hellman, S., Henderson, I.C. & Kinne, D.W. (eds) pp. 604-665. J.B. Lippincott: Philadelphia.

KAPLAN, E.L. & MEIER, P. (1958). Nonparametric estimation from incomplete observations. *J. Am. Stat. Assoc.*, **53**, 457-481.

LLUCH, A., GARCIA CONDE, J., CASADO, A., MARTIN, M., DIAZ RUBIO, E., OLIVEIRA, C., GERVASIO, M.H., DE PABLO, J.L., GARCIA GIROW, J.L., GOROSTIAGA, J., MARTINEZ, A. & DELGADO, F.M. (1992). Phase II trial with navelbine (NVB) in advanced breast cancer (ABC) previously untreated. *Proc. Am. Soc. Clin. Oncol.*, **11**, 72 (abstract 115).

MARTY, M., LEANDRI, S., EXTRA, J.M., ESPIE, M. & BESENVAL, M. (1989). A phase II study of vinorelbine (NVB) in patients (pts) with advanced breast cancer (BC). *Proc. Am. Assoc. Cancer Res.*, **30**, 256 (abstract 1017).

MATHE, G. & REIZENSTEIN, P. (1985). Phase I pharmacologic study of a new *vinca* alkaloid: navelbine. *Cancer Lett.*, **27**, 285-293.

MICKIEWICZ, E., FERNANDEZ, O., BRUNO, S., DELGADO, C., TEIXERA, L., MARTINEZ, L., LIRA-PUERTO, V., NOGUERA, C., SOLIDORO, A., OTERO, J., HEGG, R. & DELGADO, F.M. (1991). Phase II trial of navelbine (NVB) in advanced breast cancer (ABC). *Eur. J. Cancer*, **27** (Suppl. 2), S66 (abstract 368).

ROMERO, A., RABINOVICH, M.G., VALLEJO, C.T., PEREZ, R., RODRIGUEZ, R., CUEVAS, M.A., MACHIAVELLI, M., LACAVA, J.A., LANGHI, M., RORERO ACUNA, L., AMATO, S., BARBIERI, R., SABATINI, C. & LEONE, B.A. (1994). Vinorelbine as first-line chemotherapy for metastatic breast carcinoma. *J. Clin. Oncol.*, **12**, 336-341.

WHO (1979). *Handbook for Reporting Results of Cancer Treatment*. World Health Organization: Geneva.


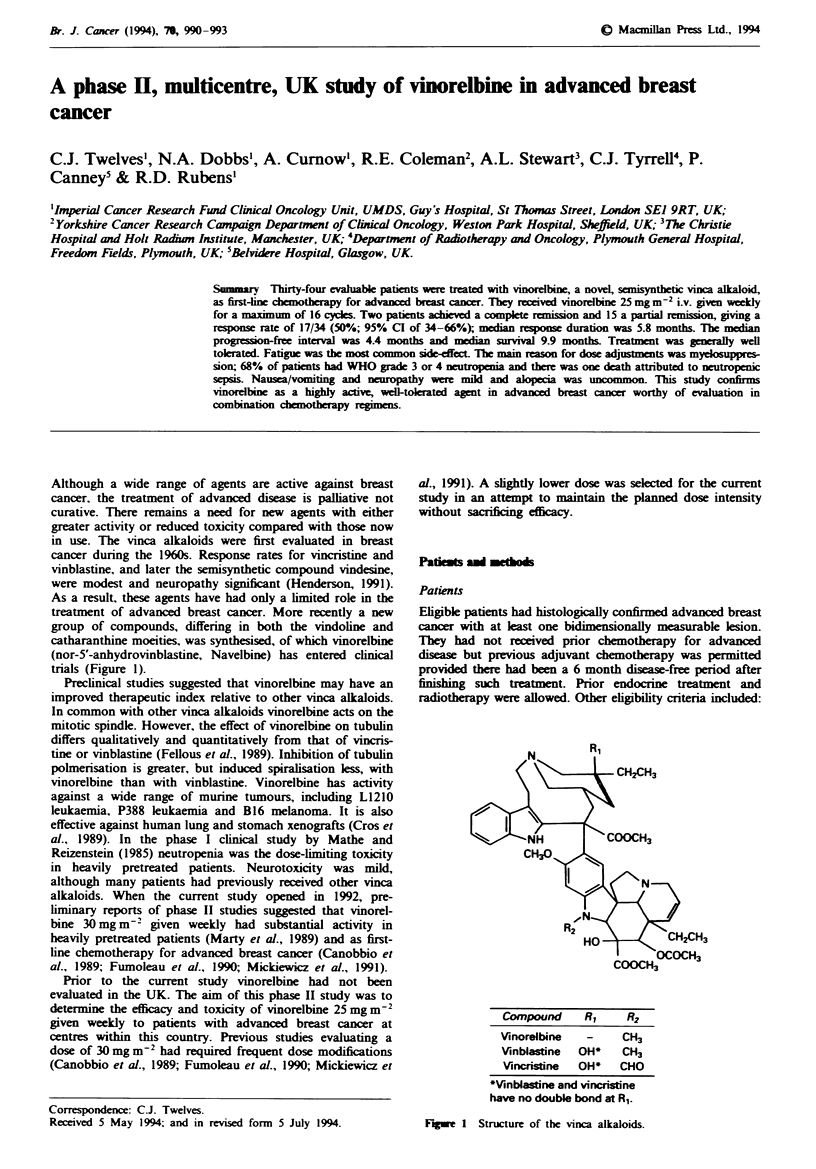

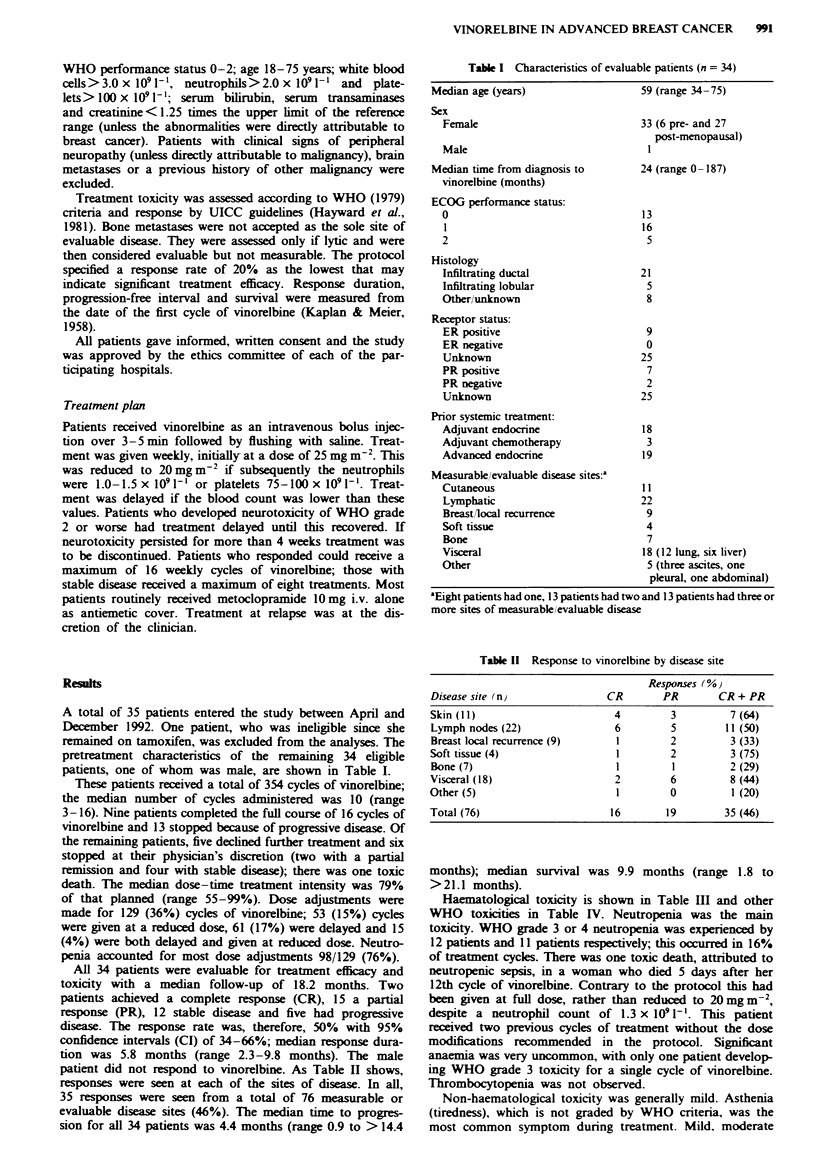

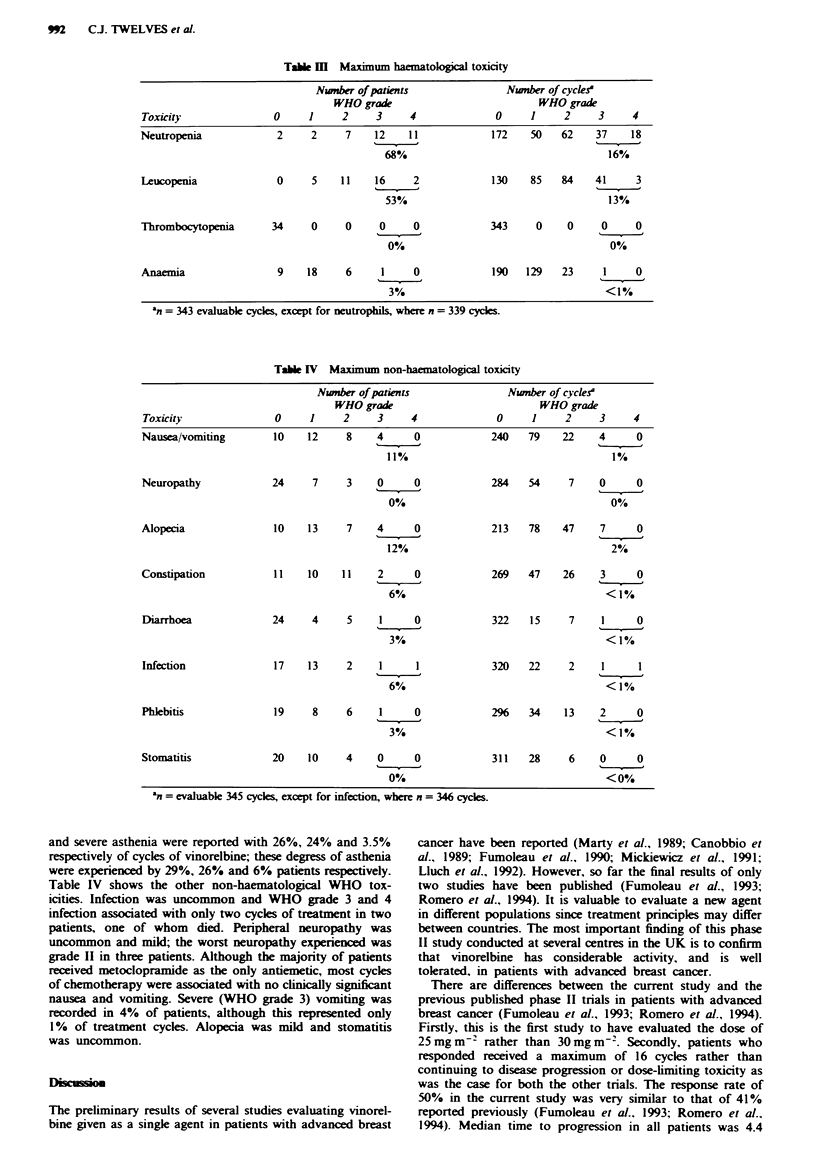

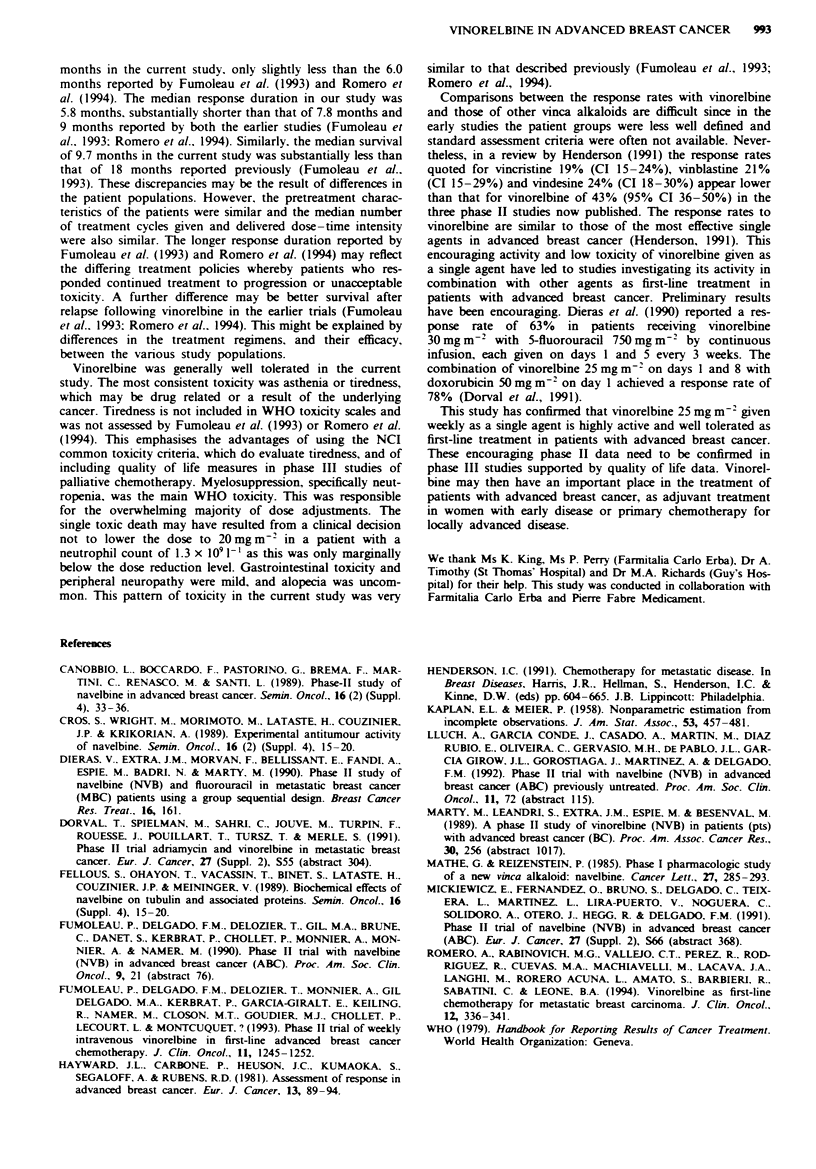


## References

[OCR_00519] Canobbio L., Boccardo F., Pastorino G., Brema F., Martini C., Resasco M., Santi L. (1989). Phase-II study of Navelbine in advanced breast cancer.. Semin Oncol.

[OCR_00546] Cros S., Wright M., Morimoto M., Lataste H., Couzinier J. P., Krikorian A. (1989). Experimental antitumor activity of Navelbine.. Semin Oncol.

[OCR_00525] Cros S., Wright M., Morimoto M., Lataste H., Couzinier J. P., Krikorian A. (1989). Experimental antitumor activity of Navelbine.. Semin Oncol.

[OCR_00551] Fumoleau P., Delgado F. M., Delozier T., Monnier A., Gil Delgado M. A., Kerbrat P., Garcia-Giralt E., Keiling R., Namer M., Closon M. T. (1993). Phase II trial of weekly intravenous vinorelbine in first-line advanced breast cancer chemotherapy.. J Clin Oncol.

[OCR_00592] Mathé G., Reizenstein P. (1985). Phase I pharmacologic study of a new Vinca alkaloid: navelbine.. Cancer Lett.

[OCR_00606] Romero A., Rabinovich M. G., Vallejo C. T., Perez J. E., Rodriguez R., Cuevas M. A., Machiavelli M., Lacava J. A., Langhi M., Romero Acuña L. (1994). Vinorelbine as first-line chemotherapy for metastatic breast carcinoma.. J Clin Oncol.

